# Impact of probiotics on gut microbiome of extremely preterm or extremely low birthweight infants

**DOI:** 10.1038/s41390-024-03520-w

**Published:** 2024-08-25

**Authors:** Lauren C. Beck, Janet E. Berrington, Christopher J. Stewart

**Affiliations:** 1 Translational and Clinical Research Institute, Newcastle University, Newcastle upon Tyne, UK; 2 Newcastle Neonatal Services, Newcastle upon Tyne Hospitals NHS Foundation Trust, Newcastle upon Tyne, UK

## Abstract

- Meta-analysis of probiotic administration to very preterm or very low birthweight (VP/VLBW) infants shows reduced risk of necrotising enterocolitis (NEC).
- Separately reported outcomes for extremely preterm infants (<28 weeks) or extremely low birth weight infants (<1000 g) (EP/ELBW) are lacking meaning some clinicians do not administer probiotics to EP/ELBW infants despite their high risk of NEC.
- We present data showing the gut microbiome is impacted in EP/ELBW infants in a similar manner to VP/VLBW infants, suggesting that risk reduction for necrotising enterocolitis that is microbiome driven will also be seen in EP/ELBW infants, making probiotic administration beneficial.

## Introduction

The use of probiotics in preterm infants has been extensively studied with at least 60 randomised controlled trials (RCTs) and 30 non-randomised studies, overall showing clinical benefit in necrotising enterocolitis (NEC) reduction by up to 50%^1,2^ Methodological issues and feeding regimes may explain variations seen with clinical use^3^ and concerns remain around practical aspects of production and use. In response to these various organisations have produced guidance/recommendations for their use including the European Society for Paediatric Gastroenterology Hepatology and Nutrition (ESPGHAN),^4^ the World Health Organisation (WHO),^5^ the American Academy of Paediatrics^6^ and the Canadian Paediatric Society.^7^ Recent intervention by the US Food and Drug Administration (FDA)^8^ after a preterm infant with birthweight <1000 g died in association with proven probiotic sepsis, and associated responses by ESPGHAN^9^ and UK physicians^10^ have once again made this a controversial area. This issue is particularly significant for the most preterm or low birthweight infants ( < 28 weeks or <1000 g), where risks of both NEC and bacterial translocation from the gut are higher compared to infants between 28 and 31 weeks, or weighing more than 1000 g.

Despite probiotics being extensively studied, much data is presented for the whole cohort <32 weeks or <1500 g, and less specifically for EP/ELBW. Although any study with inclusion criteria <32 weeks will also include a proportion of infants also <28 / < 1000 g (for instance, in SIFT (the Speed of Increasing milk Feeds Trial) this was 39%^11^) the exact proportion of EP/ELBW infants contributing to the overall meta-analysis of probiotic outcomes remains unknown. The most recent Cochrane analysis (2023) identified ten trials where some outcome measures were explicitly presented separately for infants <28 weeks gestation or <1000 g showing little or no impact on NEC (Risk Ratio (RR) 0.92, 95% Confidence Interval (CI) 0.69 to 1.22, 10 trials, 1836 infants; low certainty) in contrast to infants <32 weeks or <1500 g with NEC RR 0.54 (95% CI 0.46 to 0.65; 57 trials, 10,918 infants; low certainty).^1^

Multi-omic research may help clarify whether biological markers of probiotic efficacy are seen in the most preterm infants. We recently showed the significant and strain-dependant impact of probiotics on the gut microbiome of healthy preterm infants (all <32 weeks gestation), demonstrating that probiotic receipt was the most important driver of all co-variates.^12^ Here, we analyse samples from these infants, divided into <28 weeks and/or <1000 g (EP/ELBW) and compare them to those from infants born 29–31 weeks gestation and ≥1000 g (referred to as VP/VLBW) to help address whether probiotics differentially impact preterm infants depending on gestational age and birthweight.

## Methods

**Fig. 1:**
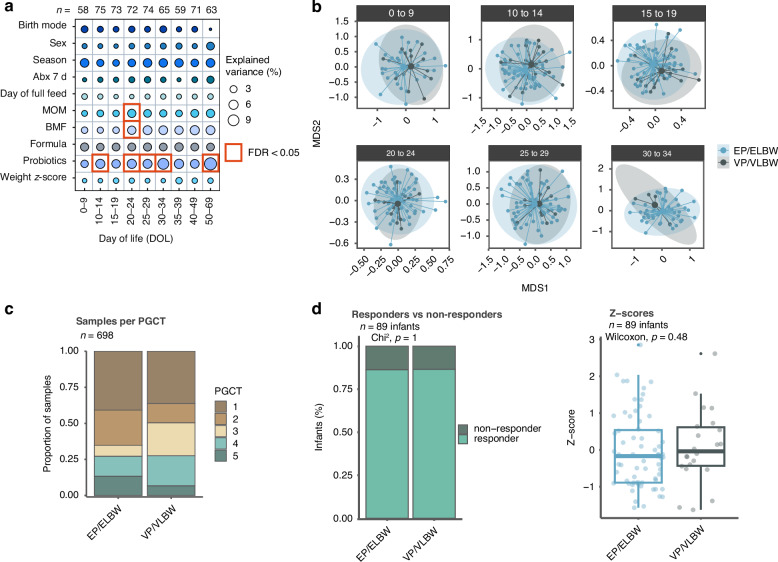
Probiotics impact the gut microbiome of EP/ELBW infants. **a** Significance and explained variance of clinical co-variates modelled by ‘adonis’ for EP/ELBW infants only. Bubbles show the amount of variance (%) explained by each co-variate at a given timepoint and significant results (FDR < 0.05) are surrounded by a red box. MOM = Mothers own milk, BMF = breast milk fortifier, Season = Spring, Summer, Autumn, Winter and antibiotics 7 days = whether the infant had received antibiotics within 7 days **b** NMDS plot of taxonomic profiles during the use of probiotics, showing the mean centroid for each group. **c** Number of samples per PGCT during probiotic use for each group. **d** Number of infants classified as responders and non-responders for each group and z scores, both based on an AUC analysis of probiotic species relative abundance during probiotic use.

For detail see Beck et al.^12^ Of the 123 < 32 weeks/<1500 g preterm infants included in the original study we identified 91 born at <28 weeks gestation and/or <1000 g leaving 32 of ≥28 weeks or ≥1000 g. Briefly the original samples and data were collected as part of a Research Ethics Committee approved study and all infants cared for in the Royal Victoria Infirmary, Newcastle, with standardised feeding, antibiotic and antifungal guidelines (prophylactic fluconazole). Between 2013 and 2016, infants received the probiotic Infloran (*B. bifidum* NCDO 2203 1 × 109 c.f.u. and *L. acidophilus* NCDO 1784 1 × 109 c.f.u.); then after mid-2016 Labinic (*B. bifidum* Bb-06 0.67 × 109 c.f.u., *B. longum* subsp. *infantis* Bi-26 0.67 × 109 c.f.u. and *L. acidophilus* NCFM 0.67 × 109 c.f.u.) was used. Stool samples were collected longitudinally (day 0 to 120), alongside extensive demographic and treatment/feed exposures. Variables fixed through time are described on a per-infant basis; other variables were categorised to reflect exposure in relation to time and therefore on a per-sample basis. DNA was extracted from ~0.1 g of stool using the DNeasy PowerSoil Kit (QIAGEN) and sequencing was performed on the HiSeq X Ten (Illumina) with a read length of 150-bp paired-end reads. Taxonomic profiling of metagenomic samples was performed using MetaPhlAn v.2.0. The five previously identified Preterm Gut Community Types (PGCTs) were used for this analysis. PERMANOVA was performed using the ‘adonis’ function and performed in cross-sectional timepoints, each with 1 sample per patient to account for repeated measures. Timepoints were selected for relevance to exposures and to give similar numbers of samples. A generalised linear mixed effects model was fit to assess whether low birthweight/gestational age (i.e. EP/ELBW vs. VP/VLBW) was associated with Shannon diversity, whilst controlling for the variables included in the beta diversity analysis and patient. Ordinations were performed using non-metric multidimensional scaling based on Bray-Curtis dissimilarity on samples collected during probiotic use. An Area Under the Curve (AUC) analysis based on the relative abundance of probiotic species during probiotic use was used to classify infants as responders or non-responders. Infants falling below 1 standard deviation from the mean, were classified as non-responders. Z scores were calculated from the same AUCs, normalised by the sampling time period for each infant. The thresholds were similar for labinic and infloran (0.1 relative abundance for Labinic infants, 0.12 for Infloran infants) and 0.1 overall and are presented combined for labinic or infloran at the combined threshold of 0.1 in Fig. 1d type.

## Results

Table 1 shows important demographics of the included 123 infants, by EP/ELBW and VP/VLBW cohorts, and relevant sample information. In total 1431 samples were analysed across 9 time points (days 0–9, 10–14, 15–19, 20–24, 25–29, 30–34, 35–39, 40–49 and 50–69) selected for relevance to exposures and to give similar numbers of samples. As in the original analysis of all <32 weeks or <1500 g^12^ probiotic receipt remained the most important identified driver of the microbiome of exclusively EP/ELBW infants (Fig. 1a).

**Table 1:** Demographic and sampling data.

	VP/VLBW	EP/ELBW
**Number of subjects (samples)**	32 (342)	91 (1089)
**Number of samples per subject**	10 (9–12)	11 (9–14.5)
**Gestational age at birth (weeks)**	29 (28–30)	26 (25–27)
**Birthweight (g)**	1315 (1170–1580)	840 (660–945)
**Day of first feed (range)**	2 (0–6)	2 (0–11)
**Day of full feeds (150mls/kg)**	12 (10–14)	14 (12–18)
**Weight z score change over study duration**	−1.3 (−2.1 to −0.5)	−1.6 (−2.1 to −0.8)
**Mothers own milk ever** ***n*** **(%)**	28 (88)	85 (93)
**Formula ever** ***n*** **(%)**	19 (59)	57 (63)
**Start day of probiotics**	6 (3–9)	7 (6–9)
**Last day of probiotics**	28 (25–35)	50 (45–61)
**Birth mode:** ***n*** **(%)**		
Caesarean	19 (59)	48 (53%)
Vaginal	13 (41)	43 (42)
**Sex** ***n*** **(%)**		
Male	24 (75)	44 (48)
Female	8 (25)	47 (52)
**Probiotic** ***n*** **(%)**		
None	9 (28)	19 (20)
Infloran	1 (3)	23 (25)
Labinic	22 (69)	49 (54)
**Season at birth** ***n*** **(%)**		
Winter	6 (19)	28 (31)
Autumn	13 (41)	28 (31)
Summer	6 (19)	25 (28)
Spring	7 (22)	20 (22)
**Antibiotics in previous 7 days** ***n*** **(%)**		
No	257 (75)	786 (72)
Yes	85 (25)	303 (28)
**Mums Own Milk at sampling** ***n*** **(%)**		
Never	38 (11)	71 (7)
During	285 (83)	878 (81)
After	19 (6)	140 (13)
**Breast Milk Fortifier at sampling** ***n*** **(%)**		
Never	184 (54)	295 (27)
Before	85 (25)	315 (29)
During	67 (20)	374 (34)
After	6 (2)	105 (10)
**Formula at sampling** ***n*** **(%)**		
Never	164 (48)	414 (38)
Before	55 (16)	334 (30)
During	98 (28)	341 (31)
After	25 (7)	0

Values are median (IQR) unless otherwise stated.

No significant differences were seen in Shannon diversity (*P* = 0.175) or overall beta diversity between EP/ELBW and VP/VLBW groups at any time point during the use of probiotics (all *P* > 0.05) (Fig. 1b). Furthermore, a similar proportion of samples from each group were classified as probiotic-associated PGCTs 4 and 5 (28% vs. 27%) (Fig. 1c). Using AUC analysis to define responders and non-responders based on the relative abundance of probiotic species, there was again no significant difference between groups (86% vs. 87% responders; *P* = 1) (Fig. 1d), reflected in the *z* score medians per group (*P* = 0.48) (Fig. 1d).

## Discussion

Microbiome differences can act as indicators of whether probiotic administration results in changes that may be mechanistically important in disease prevention.^13^ The PiPS trial^14^ that administered *Bifidobacterium breve* strain BBG-001 did not identify clinical benefit and also found no differences in gut microbiome between probiotic vs. placebo.^15^ Conversely the ProPrems trial^16^ which reported a 54% reduction in NEC in infants receiving *Bifidobacterium longum* subsp. *infantis* BB-02, *Streptococcus thermophilus* TH-4 and *Bifidobacterium animalis* subsp. *lactis* BB-12 did identify gut microbiome differences between probiotic vs. placebo.^17^ Having previously shown that in healthy preterm infants <32 weeks gestation probiotics are the dominant driver of the microbiome,^12^ we confirm here that this remains the case when considering only EP/ELBW infants. Any specific differences in the impact on the gut microbiome between the two probiotic products used are not presented here. Mechanisms by which probiotics exert their effect are variable, and some are species and strain-specific. Common or widespread microbiome-mediated effects are through competitive exclusion of other organisms or through production of beneficial metabolites (e.g. short chain fatty acids) or vitamins. Our data suggest that where probiotics do exert their effect through the microbiome it is likely that an effect seen in a cohort of VP/VLBW infants will also be seen in EP/ELBW infants.

In conclusion, although trials reporting EP/ELBW infants separately are relatively limited, we show that impacts on the infant gut microbiome seen in EP/ELBW infants are similar to those seen in VP/VLBW infants, and this should be included in decision making about probiotic administration to these infants who are at the highest risk of NEC.

## Data Availability

Suitably anonymised data may be available on reasonable request. All metagenomic sequencing data generated and analysed in the present study have been deposited in the European Nucleotide Archive under study accession no. PRJEB49383.
